# Age-dependent resistance of a perennial herb, *Aristolochia contorta* against specialist and generalist leaf-chewing herbivores

**DOI:** 10.3389/fpls.2023.1145363

**Published:** 2023-05-30

**Authors:** Se Jong Jeong, Bo Eun Nam, Hyeon Jin Jeong, Jae Yeon Jang, Youngsung Joo, Jae Geun Kim

**Affiliations:** ^1^ Department of Biology Education, Seoul National University, Seoul, Republic of Korea; ^2^ Research Institute of Basic Sciences, Seoul National University, Seoul, Republic of Korea; ^3^ The Korea National Arboretum, Pocheon, Republic of Korea; ^4^ School of Biological Sciences, Seoul National University, Seoul, Republic of Korea; ^5^ Center for Education Research, Seoul National University, Seoul, Republic of Korea

**Keywords:** aristolochic acids, C/N ratio, plant age, plant-herbivore interaction, seasonal variation, plant defense

## Abstract

Plants need to balance investments in growth and defense throughout their life to increase their fitness. To optimize fitness, levels of defense against herbivores in perennial plants may vary according to plant age and season. However, secondary plant metabolites often have a detrimental effect on generalist herbivores, while many specialists have developed resistance to them. Therefore, varying levels of defensive secondary metabolites depending on plant age and season may have different effects on the performance of specialist and generalist herbivores colonizing the same host plants. In this study, we analyzed concentrations of defensive secondary metabolites (aristolochic acids) and the nutritional value (C/N ratios) of 1^st^-, 2^nd^- and 3^rd^-year *Aristolochia contorta* in July (the middle of growing season) and September (the end of growing season). We further assessed their effects on the performances of the specialist herbivore *Sericinus montela* (Lepidoptera: Papilionidae) and the generalist herbivore *Spodoptera exigua* (Lepidoptera: Noctuidae). Leaves of 1^st^-year *A. contorta* contained significantly higher concentrations of aristolochic acids than those of older plants, with concentrations tending to decrease over the first-year season. Therefore, when first year leaves were fed in July, all larvae of *S. exigua* died and *S. montela* showed the lowest growth rate compared to older leaves fed in July. However, the nutritional quality of *A. contorta* leaves was lower in September than July irrespective of plant age, which was reflected in lower larval performance of both herbivores in September. These results suggest that *A. contorta* invests in the chemical defenses of leaves especially at a young age, while the low nutritional value of leaves seems to limit the performance of leaf-chewing herbivores at the end of the season, regardless of plant age.

## Introduction

Plants are continuously exposed to a variety of biotic and abiotic stresses in their natural environment. Herbivory is a major cause of biotic stress for plants (Mattson, 1980; Karban and Myers, 1989; Alves-Silva and Del-Claro, 2016; Iqbal et al., 2021). Plants are dynamic organisms that can optimize cost-benefit balance between growth and defense to deal with the herbivory stress (Herms and Mattson, 1992). The growth-defense trade-off of plants is based on the assumption that plants have limited resources to invest in growth and defense (Coley et al., 1985; Simms and Rausher, 1987; Huot et al., 2014). Defense mechanisms of plants against herbivory stress can vary dramatically depending on the plant’s ontogeny (Barton and Koricheva, 2010; Barton and Boege, 2017). Ontogenetic trajectories of plant defense are also generally inferred as a result of a trade-off between growth and defense, in which defense mechanisms are strengthened within specific tissues at specific ontogeny stages to achieve an optimal balance between growth and defense (Ohnmeiss and Baldwin, 2000; Barton and Boege, 2017).

There are several theoretical frameworks for interpreting how plants change their defense systems according to their ontogeny. One of them is the optimal defense theory (ODT). This theory predicts that plants should be highly defended against tissues with high fitness value or high risk of attack (McKey, 1974). And, based on ODT, plant defenses can be also variable at each stage during plant ontogeny (Ohnmeiss and Baldwin, 2000; Barton and Boege, 2017). According to ODT’s description of plant tissue defense, plants will optimize themselves against herbivory based on fitness values of various tissues (e.g., flowers) and the likelihood that those tissues would be attacked (e.g., young leaves) (Coley, 1983; Ohnmeiss and Baldwin, 2000; Alba et al., 2012). Similar to this, when describing defense in accordance with the plant’s ontogenic stage as ODT, plants might have a high level of defense in the reproductive stage when it has the highest fitness (Boege, 2005) and in the juvenile stage due to its greater susceptibility to herbivore attack than older stages (Bryant et al., 1991). Therefore, it is difficult to generalize because the ontogenetic trajectory for the defense of plants differs by species.

Many studies confirming the ontogenetic trajectory for the defense of herbaceous plants have been mainly conducted on annual plants, which have relatively rapid life cycles (Barton and Koricheva, 2010; Barton and Boege, 2017). Perennial plants can maintain different sequential life-history strategies based on their age and physiological and ecological conditions in the previous year (Bryant et al., 1991; Iwasa, 2000). For annual plants, variation in ontogeny is closely linked to variation in phenology. However, for perennial plants, phenological variation might be more independent of ontogenetic variation. That is, it will be especially crucial to understand relationships between within-year phenological variance and among-year ontogenetic variation for perennial plants (Yang et al., 2020; Cope et al., 2022). Therefore, age- and season-dependent variations of plants can be main factors to understand the trajectory of defense change in perennial herb.

Herbivore performance depends on their ability to deal with the host plant defenses. It varies greatly depending on the herbivore’s level of specialization on host plants (Agrawal, 2007; Ali and Agrawal, 2012; Thiel et al., 2020). Specialist herbivores (mono- or oligophagous) mainly feed on their host plant family since they are able to detoxify and/or sequester specific secondary metabolites of their host plants (Agrawal, 2007; Lankau, 2007; Ali and Agrawal, 2012; Thiel et al., 2020). That is, specialist herbivores are considered to be the result of co-evolution with their host plants (Karban and Agrawal, 2002; Ali and Agrawal, 2012; Maron et al., 2019). The variability of plant’s defense level can have different levels of impact on the performances of specialist and generalist herbivores. However, many studies have focused primarily on the age of the leaf itself (Ernest, 1989; Gutbrodt et al., 2012; Cao et al., 2018). Based on the fact that defense levels of perennial plants can change depending on plant age and season (Yang et al., 2020), it is necessary to conduct empirical research on how variable defense levels of perennial herbs based on plant age and season can affect performances of specialist and generalist herbivores occupying the same host plant.

Plant defenses can be divided into mechanical and chemical defenses traditionally. Plant mechanical defenses (e. g. lignified plant cell wall, trichome density, cuticle thickness) can be variable to biotic and abiotic stress (Moura et al., 2010; Kuglerová et al., 2019; Saska et al., 2021) and can also be variable depending on plant ontogeny (Kearsley and Whitham, 1998; Traw and Feeny, 2008; Barton and Koricheva, 2010). However, we focused on the plant chemical defenses in this study given that *Aristolochia contorta* Bunge synthesizes specific toxic secondary metabolites. *Aristolochia contorta* is a herbaceous perennial vine distributed near the edge of forests or rivers in East Asia (Nakonechnaya et al., 2012). It has been observed that its sexual reproduction only occurs in 3-year-old or older plants (Park et al., 2019). *Aristolochia contorta* can synthesize specific toxic secondary metabolites including aristolochic acids (Cheung et al., 2006). Such aristolochic acids can negatively affect generalist herbivore’s growth at low concentrations (Jeude and Fordyce, 2014) and could be key factors in the defense of *A. contorta* against herbivores. However, concentrations of aristolochic acids induced by simulated herbivory were not high enough to affect performance of the specialist herbivore (Park et al., 2022). Biosynthetic pathway of aristolochic acids in Aristolochiaceae family is still partly revealed (Cui et al., 2022). *Sericinus montela* (dragon swallowtail) is known as a specialist herbivore of *A. contorta* and a vulnerable species in the Red List of the Republic of Korea (National Institute of Biological Resources, 2012).

The ontogenetic trajectory of defensive secondary metabolites is still controversially discussed. Barton and Koricheva (2010) reported that concentrations of defensive secondary metabolites in herbaceous plants tend to increase during life, while Yang et al. (2020) observed a decline in chemical defense along the ontogenetic trajectory. We hypothesized that: (1) the growth and nutrient composition (C/N ratio) of *A. contorta* differ by plant age and season; (2) the concentrations of aristolochic acids in *A. contorta* tissues differ by plant age and season; (3) the performance of specialist and generalist herbivores is influenced by differences in the defense of *A. contorta* depending on plant age and season. To test these hypotheses, we grew one, two- and three-year-old *A. contorta* individuals in an outdoor growing facility. In addition, growth parameters, aristolochic acids concentrations, and C/N ratio of each plant organ were quantified in July (the middle of growing season) and September (the end of growing season). Finally, leaf feeding trials were conducted on specialist (*S. montela)* and generalist herbivores (*Spodoptera exigua*) to assess effects of age-dependent plant defense traits on herbivore performances. Our findings will contribute to our understanding of plant-herbivore interaction dynamics along the life history of perennial herbs.

## Material and methods

### Plant material

*Aristolochia contorta* seeds were collected at Pyeongtaek (37°05’43” N, 127°05’15” E) and Gapyeong (37°34’54’’ N, 127°31’41’’ E), Gyeonggi-do, South Korea, in 2018, 2019, and 2020. Collected seeds were stored in a refrigerator at 4°C under dry conditions and sown the following year to obtain one, two, and three-year-old plants for the study. Germinated seedlings were transplanted into pots (25 cm in diameter and 25 cm in depth). Mixed soil considering the soil texture of its natural habitat (sand:topsoil = 2:1, v/v) was used for sowing and transplanting (Park et al., 2019). All individuals were grown in an experimental plot in Seoul National University (37°27’49” N, 126°57’19” E) covered with mesh for 50% of relative light intensity, with which *A. contorta* showed more vigorous growth rather than that with open canopy (Park et al., 2019). We regularly fertilized every pot using a proper quantity based on the pot’s volume (Park et al., 2021; Park et al., 2022). At the harvest stage, which will be described later, populations of Pyeongtaek and Gapyeong were reasonably and evenly distributed across each plant age group (*n* = 21 and 19 in 1^st^-year; *n* = 17 and 23 in 2^nd^-year; *n* = 17 and 23 in 3^rd^-year, respectively).

### Growth measurement of plant

Height was measured and number of leaves were counted weekly or biweekly for 12 representative plants of each age group during the growing season of 2021. This was the period from 10 days after the first sprouting until 15 September, when the above-ground growth stopped. Plants from each group (*n* = 20 for each group) were harvested in July (the middle of growing season) and September (the end of growing season). They were divided by plant organ into leaves, stems, and roots, and fresh weights were measured. Total stem length of each harvested individual was also measured. Total leaf area was measured using a portable leaf area meter LI-3000C and a transparent belt conveyer LI-3050C (LI-COR, NE, USA). After leaf area measurement, the 5^th^ to 7^th^ leaves from the uppermost leaf were taken to standardize the leaf samples for the analysis of secondary metabolites (*n* = 10) and C/N ratio (*n* = 7) (Park et al., 2021; Park et al., 2022). The leaf samples destined for the quantification of aristolochic acids were weight. Stems and roots were subsampled at approximately 100 mg from each plant. The remaining plant material was dried in a dry oven at 60°C over three days. Dry weight was then measured. We estimated the moisture content for each plant age and organ based on the fresh and dry weights. Dry weight loss due to subsampling was corrected for moisture content of fresh weight of subsampled organs. However, in the case of 1^st^-year plants, the mass of samples for metabolite analysis accounted for a large portion of the plant. Thus, the dry mass of 1^st^-year plants was measured from individuals that had not been subsampled (*n* = 10).

### Carbon and nitrogen analysis of plant

To calculate the C/N ratio, we performed stoichiometric analyses of total carbon (TC) and total nitrogen (TN) ratios in plant organs. Subsampled organs were dried at 60°C and ground (Wiley Mini-Mill 3380L10, Thomas Scientific, NJ, USA) to make homogeneous mixtures. Total carbon and TN were determined using an elemental analyzer (Flash EA 1112, Thermo Electron, USA) at Seoul National University’s National Instrumentation Center for Environmental Management.

### Quantification of plant specific secondary metabolites

Secondary metabolites were extracted using the methods of Park et al. (2021) with minor modifications. First, samples of organs from each group of plants were frozen and ground in liquid nitrogen. Frozen materials (approximately 100 mg) were extracted by adding 1 ml of 80% MeOH (HPLC grade) and then homogenized with a genogrinder (1600 MiniG; SPEX Certi Prep, NJ, USA) operated at 1,200 strokes per min for 120 sec. Supernatants were collected after centrifugation at 16,100 g for 20 min at 4°C. Then 850 μl of the supernatant was transferred to a new 1.5 ml tube. After drying the solvent in a speedvac (500 rpm, c.a. 120 min; HyperVAC VC2124; Hanil Scientific, Daejeon, Republic of Korea) until dryness, the dried pellet was resuspended with 250 μl of 40% MeOH (HPLC grade) using a vortex mixer for 10 min. After removing unsolved particles using syringe filters (0.22 μm) and centrifugation at 16,100 g for 20 min at 4°C, 150 μl of particle-free supernatant was transferred to a chromatography vial.

Quantification was conducted using an HPLC-DAD (Dionex Ultimate 3000; Thermo Scientific, MA, USA) following the method of Araya et al. (2018) with minor modifications. Standard solutions of aristolochic acids 1 and 2 (Sigma-Aldrich, MO, USA) were separately prepared at concentrations of 100, 10, 1, 0.1, and 0.01 μg/ml and diluted in 40% MeOH. A UHPLC C18 column (2.1 mm x 100 mm, 1.7 μm particle size, ACQUITY UPLC CSH C18 Column, Waters, MA, USA) was used for the analysis coupling with a C18 pre-column (2.1 mm x 5 mm, 1.7 μm particle size, ACQUITY UPLC CSH C18 VanGuard Pre-column, Waters, MA, USA). The column was maintained at 40°C. Mobile phase consisting of distilled water with 0.1% acetic acid (A) and acetonitrile with 0.1% acetic acid (B) was pumped at a flow rate 0.4 ml/min. Gradient elution program was as follows: 0 – 1.74 min, 0% B; 1.74 – 12.16 min, 0 – 20% B; 12.16 – 12.5 min, 20 – 50% B; 12.5 – 17.72 min, 50% B; 17.72 – 18.06 min, 50 – 100% B; 18.06 – 18.41 min, 100 – 100% B; 18.41 – 19.1 min, 100 – 0% B; and 19.1 - 19.5 min, 0% B. The injection volume was 3 μl. The detection was performed with a diode array detector (DAD), recording spectra between 190 and 400 nm. Peak intensities of aristolochic acids 1 and 2 were quantified at 254 nm. Using reaction curves of a standard solution, exact concentrations of aristolochic acids 1 and 2 of samples were determined.

### Quantification of herbivores performance

Leaf feeding trials were conducted in July and September, which corresponded to the stoichiometric analysis period. Eggs of *S. montela* were collected on 14^th^ of July and 9^th^ of September in 2021, respectively. Larvae hatched on July 16 and September 13, respectively. They were raised with enough *A. contorta* leaves and space to ensure that all larvae were in the second instar stage. At this time, *A. contorta* leaves supplied with water and kept fresh, and consisted of leaves of plants older than 4 years. When larvae reached second instar stage (July 19 and September 17, respectively), 5^th^ to 7^th^ leaves were collected from the uppermost leaf of 1^st^-year, 2^nd^-year, and 3^rd^-year plants. To prevent drying, the petiole of each leaf was immersed in water in a 1.5 mL tube and sealed with Parafilm. Aluminum foil was used to make tube supports, allowing larvae to move freely on leaves (Park et al., 2021). A total of 60 specialist larvae (about 4 mg) were used for each leaf feeding trial. Randomized sets of 20 larvae were used for each group. Each larva was separately placed in a breeding dish (90 mm diameter × 40 mm height, cap with nylon-mesh). Whenever more than 50% of leaves were consumed, leaves were replaced with new ones. We took a photo of every leaf before and after treatment to calculate the consumed leaf area using ImageJ (Schneider et al., 2012). This leaf feeding trial was performed for six days. Relative growth rate (RGR) of individual larva was calculated as the ratio of daily weight gain to initial weight. Estimated assimilation rate was calculated as the ratio of daily weight gain from the estimated leaf weight consumed. Because the ratio of leaf weight to leaf area might vary with plant age, leaf weights were calculated based on leaf area per plant age according to the equations (**Figure S1**).

*Spodoptera exigua* larvae are known to feed on *A. contorta* leaves in the field (Park et al., 2022). To evaluate generalist herbivore performance, the eggs of *S. exigua* were purchased from a laboratory colony of the Biological Utilization Institute (Andong-si, Gyeongsangbuk-do, Republic of Korea). A total of 180 second instar larvae of *S. exigua* were selected, with 60 larvae per experimental treatment, due to higher lethality compared to the specialist herbivore. For each experimental treatment, three larvae were placed in a single breeding dish with an individual leaf. All other details of the leaf feeding trial were identical to those of *S. montela*. Leaf feeding trials for generalist herbivore were also started on 22^th^ of July and 28^th^ of September in 2021, respectively, to match the time period used for leaf feeding trial of specialist herbivore. During leaf feeding trials, we maintained the breeding temperature at 24°C for both July and September.

### Statistical analysis

Two-way analysis of variance (ANOVA) was conducted for plant age (1^st^-year, 2^nd^-year, and 3^rd^-year) and season (July and September). Duncan’s post-hoc test was used to examine statistical differences between experimental conditions. All tests were performed using SPSS 25.0 software (SPSS, Inc., Chicago, IL, USA). Significance level was set at *p* < 0.05.

## Results

### Plant growth and C/N ratio

**Figure 1:**
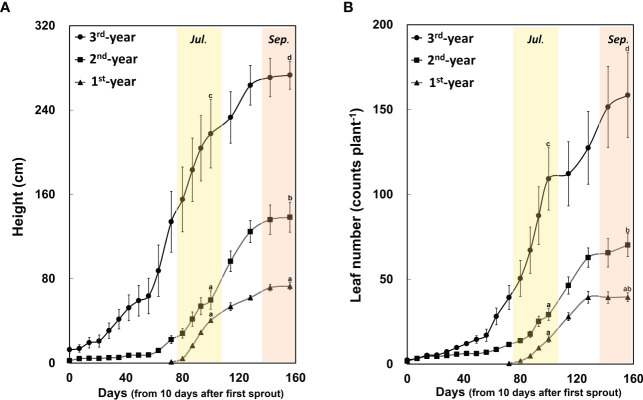
Height **(A)** and leaf number **(B)** of *Aristolochia contorta* increased with plant age. They were measured from April 12, 2021 (*n* = 12, respectively). Two-way ANOVA was conducted for plant height and leaf number according to plant age and season as of July 21 (the growing season) and September 15 (the end of the growing season). Vertical bar indicates standard error for each group. Different letters represent statistically different sub-groups by Duncan’s post-hoc test (*p* < 0.05).

**Figure 2:**
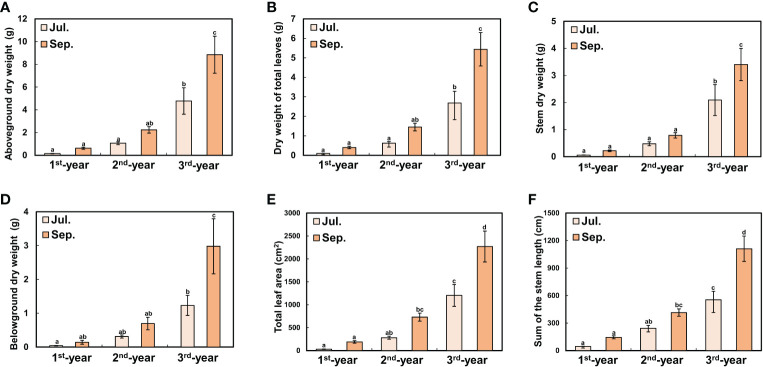
Growth degree of *Aristolochia contorta* was measured on July 29 (the growing season) and September 30 (the end of growing season). **(A)** Total aboveground dry weight, **(B)** Dry weight of total leaves, **(C)** Stem dry weight, **(D)** Belowground dry weight, **(E)** Total leaf area, and **(F)** Sum of stem length for each group increased with plant age and season. **(A-D)** Vertical bar indicates standard error for each group (*n* = 10 in 1^st^-year plant; *n* = 20 in 2^nd^-year plant and 3^rd^-year plant). **(E, F)** Vertical bar shows standard error for each group (*n* = 20, respectively). Different letters represent statistically different sub-groups by Duncan’s post-hoc test (*p* < 0.05).

**Figure 3:**
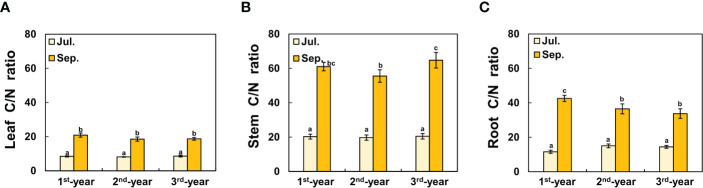
C/N ratio of each organ of *Aristolochia contorta* in September was more than doubled than that in July, which was statistically significant, although there was no statistically significant difference in plant age. **(A)** Leaf C/N ratio, **(B)** Stem C/N ratio, **(C)** Root C/N ratio. The vertical bar shows standard error for each group (*n* = 7, respectively). Different letters represent statistically different sub-groups by Duncan’s post-hoc test (*p* < 0.05).

Between 1^st^-year plants and 2^nd^-/3^rd^-year plants, sprouting time showed a difference of about two months (2^nd^-/3^rd^-year *A. contorta* started to sprout in early April and 1^st^-year *A. contorta* sprouted in early June). *Aristolochia contorta* individuals of each age group gradually increased in height and number of leaves over time. Such increases were evident in June and July, the growing season of the plant (**Figure 1**). Finally, plant growth such as height and number of leaves almost stopped in September in all groups (**Figure 1**). Dry weight of each organ of *A. contorta* increased with the passage of season and year (**Figures 2A-D**). The C/N ratio for each organ of the plant was higher in September than in July (**Figure 3**). However, there was no statistically significant difference in the C/N ratio for each organ of the plant according to age (**Figure 3**; **Table 1**).

**Table 1:** *F*-values and *p*-values from two-way analysis of variance (ANOVA) for effects of plant age and season on growth, C/N ratio, aristolochic acid concentrations of *A. contorta*, and herbivores performance (statistically significant effects are presented in boldface).

		Age		Season		Age × Season	
		*F*	*p*	*F*	*p*	*F*	*p*
Growth	Height (cm)	80.996	0.000	20.880	0.000	0.615	0.544
	Leaf number (individual^-1^)	36.924	0.000	12.858	0.001	0.459	0.634
	Aboveground dry weight (g)	26.783	0.000	6.480	0.013	3.001	0.055
	Dry weight of total leaves (g)	29.243	0.000	8.403	0.005	3.119	0.049
	Dry weight of stem (g)	21.655	0.000	2.906	0.092	1.189	0.909
	Belowground dry weight (g)	30.588	0.000	32.121	0.000	21.202	0.000
	Total leaf area (cm^2^)	48.028	0.000	15.612	0.000	3.573	0.031
	Sum of the stem lenght (cm)	55.674	0.000	22.373	0.000	5.913	0.004
C/N ratio	Leaf	1.457	0.246	270.727	0.000	1.227	0.305
	Stem	1.604	0.215	312.530	0.000	1.157	0.326
	Root	1.228	0.305	237.383	0.000	5.324	0.009
Aristolochic acid 1 (ng mg^-1^)	Leaf	49.979	0.000	6.040	0.017	5.246	0.008
	Stem	44.093	0.000	9.825	0.003	26.198	0.000
	Root	0.901	0.412	28.512	0.000	0.603	0.551
Aristolochic aicd 2 (ng mg^-1^)	Leaf	5.967	0.005	0.810	0.372	0.219	0.804
	Stem	12.721	0.000	5.686	0.021	9.949	0.000
	Root	0.503	0.608	21.285	0.000	0.248	0.781
Herbivore performance	Relative growth rate of *S. montela* (day^-1^)	3.718	0.027	305.173	0.000	8.952	0.000
	Consumed leaf area of *S. montela* (cm^2^)	0.236	0.790	196.949	0.000	6.219	0.003
	Assimilation rate of *S. montela*	0.049	0.954	237.548	0.000	0.354	0.702
	Relative growth rate of *S. exigua* (day^-1^)	2.451	0.095	14.017	0.000	3.497	0.067
	Consumed leaf area of *S. exigua* (cm^2^)	3.812	0.028	16.895	0.000	1.102	0.298
	Assimilation rate of *S. exigua*	0.386	0.682	1.143	0.290	0.054	0.817

### Aristolochic acids concentrations by plant age and season

**Figure 4:**
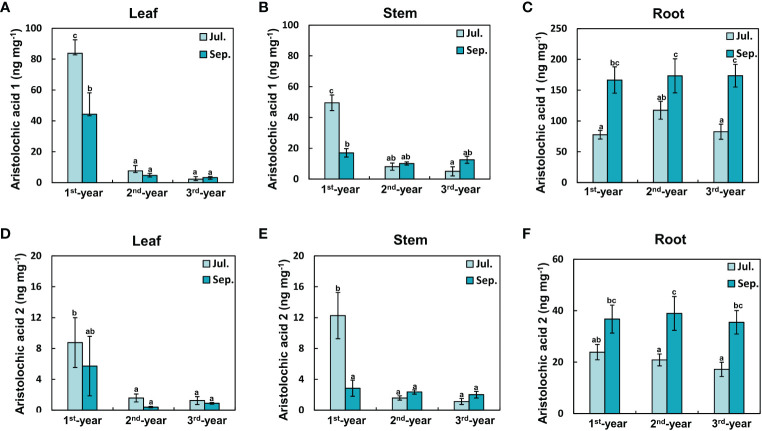
Concentrations of aristolochic acids in each plant organ were different with plant age and season. **(A)** Leaf aristolochic acid 1, **(B)** Stem aristolochic acid 1, **(C)** Root aristolochic acid 1, **(D)** Leaf aristolochic acid 2, **(E)** Stem aristolochic acid 2, **(F)** Root aristolochic acid 2. **(A, B, D, E)**: In leaves and stems of plants, concentrations of aristolochic acids 1 and 2 were the highest in July of the 1^st^-year plant. They decreased markedly with age. **(C, F)**: In roots of plants, concentrations of aristolochic acids 1 and 2 increased more in September than in all other groups. The vertical bar shows standard error for each group (*n* = 10; in case of 1^st^-year plant roots, *n* = 9). Different letters represent statistically different sub-groups by Duncan’s post-hoc test (*p* < 0.05).

Concentrations of aristolochic acids 1 and 2 in leaves and stems decreased with the increasing age of plants (**Figures 4A, B, D, E**). In particular, concentrations of aristolochic acid 1 in 1^st^-year leaves in July were significantly higher than those in 2^rd^-year and 3^rd^-year leaves (1^st^-year, 83.80 ± 8.69 ng mg^-1^; 2^rd^-year, 7.61 ± 4.62 ng mg^-1^; 3^rd^-year, 2.26 ± 1.61 ng mg^-1^). Concentration of aristolochic acid 1 in September in 1^st^-year leaves decreased to almost half of that in July (July, 83.80 ± 8.69 ng mg^-1^; September, 44.29 ± 13.88 ng mg^-1^). As such, leaf aristolochic acid 1 concentrations were significantly related to both individual and combined effects of plant age and season (**Figure 4A**; **Table 1**). Concentrations of aristolochic acids 1 and 2 were much higher in the root than in the leaf or stem regardless of the season (**Figure 4**). They showed statistically significant differences according to the season but not according to plant age (**Table 1**). That is, concentrations of aristolochic acids in roots tended to be higher in September than in July. This trend was somewhat opposite to that in leaves (**Figures 4C, F**). In addition, concentrations of aristolochic acids in plant leaves and roots were not statistically different across source populations (**Figure S2**).

### Herbivore performance by plant age and season

**Figure 5:**
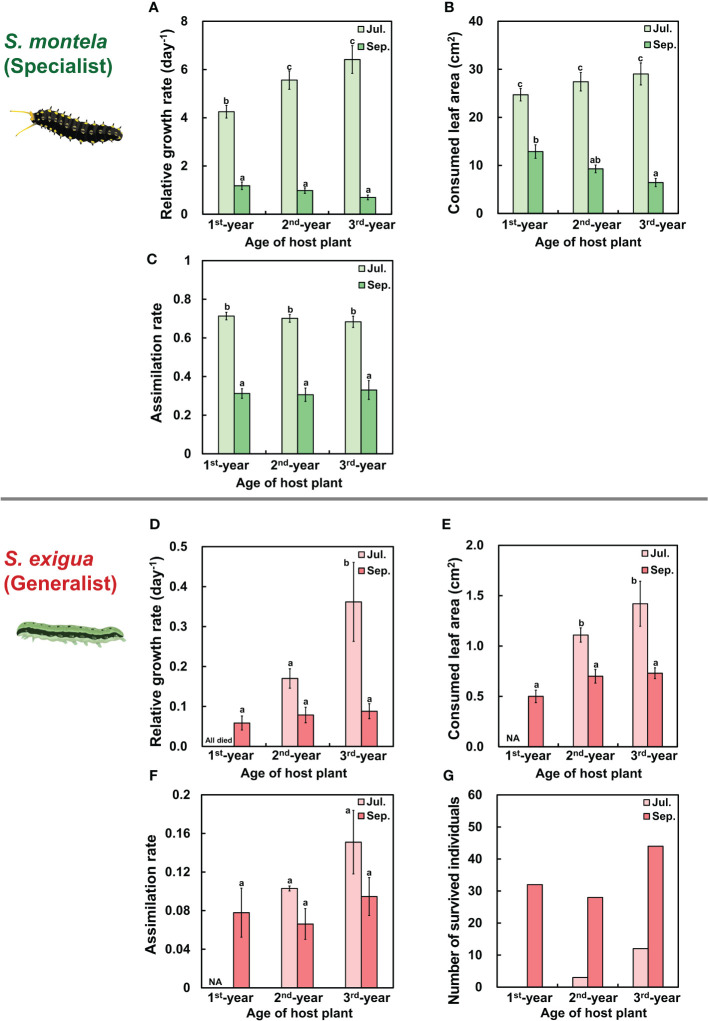
Performances of specialist and generalist herbivores were differently affected by plant age and season. **(A, D)** Relative growth rate (RGR), **(B, E)** Consumed leaf area by herbivores, **(C, F)** Estimated assimilation rate in **(A–C)** *Sericinus montela* (specialist herbivore) and **(D–F)** *Spodoptera exigua* (generalist herbivore). **(G)** Number of survied individuals of *S. exigua* (generalist herbivore). Note that when larvae were fed the 1^st^-year plant in July, the specialist did not grow well and generalists were wiped out. The vertical bar in **(A–C)** shows the standard error for the number of survived individuals in each group (*n =* 20; except 3^rd^-year in July, *n* = 19; 3^rd^-year in September, *n* = 18). Vertical bar in **(D–F)** shows standard error for each group (*n =* based on **(G)** number of survived individuals). Different letters represent statistically different sub-groups by Duncan’s post-hoc test (*p* < 0.05).

We examined the effects of plant age and season on the performances of herbivores by measuring relative growth rates, consumed leaf areas, and estimated assimilation rates of *S. montela* (specialist herbivores) and *S. exigua* (generalist herbivores). Overall, relative growth rate, consumed leaf area, and assimilation rate showed similar patterns (**Figure 5**). In particular, *S. montela* larvae in July showed the lowest growth rate in the 1^st^-year *A. contorta* leaves feeding group among three plant age feeding groups, with differences among groups being statistically significant (**Figure 5A**; **Table 1**). However, there was no statistically significant difference in consumed leaf area or assimilation rate according to plant age (**Figures 5B, C**; **Table 1**). In September, the relative growth rate of larvae in all groups decreased dramatically, although larvae were kept at the same temperature as in July. Such decreases were also statistically significant (**Figures 5A–C**; **Table 1**).

All larvae of *S. exigua* died when they were fed with 1^st^-year *A. contorta* leaves in July, which was the only case among all age and season groups (**Figures 5D**). Their survival number was the highest in the 3^rd^-year feeding group, followed by that in the 2^nd^-year feeding group (**Figures 5D, G**). When comparing 2^nd^-year and 3^rd^-year feeding groups in July, *S. exigua* larvae in the 3^rd^-year *A. contorta* leaf feeding group showed a higher relative growth rate than those in the 2^nd^-year feeding group (**Figures 5D**). The relative growth rate of *S. exigua* larvae decreased in September compared to July in the 2^nd^-year and 3^rd^-year feeding groups (**Figures 5D**). However, the number of survived individuals for six days was significantly higher in September than in July (**Figure 5G**).

## Discussion

Our findings show that the level of investment of perennial herbs in growth and defense can change with plant age and season and with plant organs, showing differential impacts on specialist and generalist herbivores. The aboveground biomass of *A. contorta* increased with age and season (**Figures 1**, **2**), with larvae having a large amount of available food from older plants. The C/N ratio is widely regarded as a measure of plant nutritional value. A high C/N ratio of a plant means that it has a low nutritional value for herbivores (Mattson, 1980; Pérez-Harguindeguy et al., 2003; Couture et al., 2010). In other words, considering C/N ratios of plants, the amount of food plants might be higher in September than in July, but the nutritional value of the food plants might be higher in July than in September (**Figure 3**). The higher C/N ratio in plant leaves in September than in July could be interpreted as an increase in carbon that allows carbon-based structural constituents to inhibit with assimilation of herbivores (Royer et al., 2013). While total carbon was also increased in the present study, the decrease in total nitrogen was relatively more pronounced (**Figure S3**). This seasonal decline in leaf N concentration is a common phenomenon of leaf senescence (Aerts, 1996; Franklin and Agren, 2002; Habekost et al., 2008; Li et al., 2017) and the relative N reduction in leaves can act as a nutritional limitation to leaf-chewing herbivores.

Aristolochic acids have a drastic effect on generalist herbivores, even in small doses (Jeude and Fordyce, 2014). Thus, a decrease in the concentration of aristolochic acids in *A. contorta* leaves with age indicates a decrease in the level of constitutive chemical defenses in aged plants (**Figures 4A, D**). However, the question remains as to where the plant invests its energy after the 1^st^-year of germination. In this regard, we offer two explanations. First, after the 1^st^-year, *A. contorta* can invest more in growth than in chemical defense. Plants can selectively invest their limited resources to their priorities between growth and defense (Coley et al., 1985; Simms and Rausher, 1987; Herms and Mattson, 1992). In general, young plants may suffer greater damage from herbivory due to their lower biomass even if they have the same herbivory intensity (Bryant et al., 1991; Dirzo et al., 2007; Boege et al., 2011). Thus, 1^st^-year *A. contorta* can minimize damage from herbivores by investing in chemical defense, whereas older *A. contorta* will invest in growth more than in defense. The second explanation is that defensive traits of *A. contorta* might change over years. Ontogenetic changes of a plant’s defense traits are frequently interpreted as changes from direct to indirect, from constitutive to induced, and from resistance to tolerance (Boege, 2005; Barton and Boege, 2017). In particular, plant’s tolerance is likely to be higher in mature plants than in young plants because of limited resource acquisition and storage reserves in young plants (Strauss and Agrawal, 1999; Hanley and Fegan, 2007). However, since changes in defensive traits based on plant ontogeny are hard to be generalized to all plant species (Goodger et al., 2013; Barton and Boege, 2017; Ochoa-López et al., 2020), it is necessary to further study whether *A. contorta* has other defense traits besides constitutive and chemical defenses with age.

Roots of *A. contorta* contained more aristolochic acids than leaves (**Figures 4A, C, D, F**), consistent with results of Cui et al. (2022). The high concentration of aristolochic acids in the root does not seem to be that the chemical defenses of *A. contorta* are focused only on the leaves. There may be unidentified antagonists in the root zone against which aristolochic acids in the root provide protection. This is supported by the observation that the concentrations of aristolochic acids in the aboveground part decreased significantly after the first year, while concentrations in the belowground parts remained high regardless of plant age. In addition, it is also noteworthy that there was a seasonal increase in aristolochic acids in the roots from July to September (**Figures 4C, F**). In fact, it has been found that *A. contorta* has a low genetic diversity because it relies heavily on asexual reproduction, including vegetative propagation by roots, rather than sexual reproduction (Nakonechnaya et al., 2012; Nam et al., 2020). And, when *A. contorta* started sexual reproduction, which was in the third year in our experiment, the concentration of aristolochic acid 1 increased in flowers and fruits over the course of the season (**Figure S4**), but nonetheless concentrations of aristolochic acids in the roots remained higher in September than July. According to the optimal defense theory, plants should have strong defenses in their stages or tissues that have a high fitness value or a high risk of attack (McKey, 1974; Boege, 2005). Thus, *A. contorta* might have allocated aristolochic acids for survival and asexual reproduction in the following year by accumulating metabolites in their roots at the end of growth.

This age-dependent defensive traits of *A. contorta* differentially affected growths of *S. montela* (specialist herbivores) and *S. exigua* (generalist herbivores). In July, *S. montela* larvae given 1^st^-year *A. contorta* leaves showed the lowest growth rate among three plant age groups (**Figure 5A**). This result is presumably due to high concentrations ofaristolochic acids in host plant leaves (**Figures 4A, D**). However, in terms of survival of all larvae in the 1^st^-year plant-fed group, the specialist herbivore showed a high ability to overcome defenses by specific secondary metabolites, aristolochic acids. Specialist herbivores can detoxify or sequester specific secondary metabolites (Agrawal, 2007; Lankau, 2007; Ali and Agrawal, 2012), but we do not yet know the specific mechanism by which *S. montela* detoxifies aristolochic acids. But, *S. montela* may also sequester aristolochic acids, as has been reported in other *Aristolochia*-feeding swallowtail butterflies (Sime et al., 2000; Fordyce et al., 2005). This is also suggested by the fact that *S. montela* larvae possess an osmeterium known to contain high concentrations of aristolochic acids in the secretory fluid of *Aristolochia*-feeding larvae of *Battus polydamas* (Priestap et al., 2012).

On the other hand, generalist herbivores “generally” detoxify and suppress plant defense mechanisms and might have a greater impact than specialists on growth and survival by fluctuating plant defense levels (Ali and Agrawal, 2012; Thiel et al., 2020). Generalist herbivores are highly susceptible even to low aristolochic acid concentrations (Jeude and Fordyce, 2014; Park et al., 2022). Therefore, the fact that the survival number of *S. exigua* larvae in July was the highest in the 3^rd^-year feeding group but the lowest (all died) in the 1^st^-year feeding group (**Figure 5D, G**) showed that 1^st^-year *A. contorta* could more effectively defend against generalist herbivores than against specialist herbivores.

The relative growth rates of both herbivore larvae were significantly lower in September than in July, with the exception of *S. exigua* larvae on one-year-old plants (all died in July), despite similar experimental temperatures (**Figure 5D**). This pattern was reflected in correspondingly lower consumed leaf area and assimilation rates in September (**Figures 5E, F**). This result might come from decreased food quality (significantly lowered nitrogen content) which could be referred from a high C/N ratio (**Figure 3A**; **Figure S3D**). A relatively low nitrogen content of a host plant might lengthen the development period of lepidopteran larvae that feed on it and lower their relative growth rate (Mattson, 1980; Fischer and Fiedler, 2000; Han et al., 2014). Thus, it could be assumed that the amount of leaves consumed was actually decreased because the development itself, including the relative growth rate, was slow in September after consuming low-nitrogen leaves. *S. montela* larvae are observed in natural habitats even at the end of September, but most of them prepare for overwintering in pupa stage, rather than adults coming out later (Kim and Kwon, 2010). This is usually attributed to lower temperatures. However, in September, even in greenhouses with controlled warm conditions, we observed delayed larval development and pupation in the fourth rather than the fifth instar stage (personal observation by SJJ and JYJ), suggesting that larval development may be delayed by a decrease in the nutritional value of the host plant. Since *S. montela* has many natural enemies mainly in the larval stage (Shin, 1974), delayed larval development on plants with low nutritional value may lower the fitness of the population in its natural habitat. In addition, *S. exigua* showed higher survival rate in September than in July regardless of the plant age (**Figure 5G**). This result is also presumed to be because leaf consumption of *S. exigua* decreased in September (**Figure 5F**) due to a higher C/N ratio in leaves in September than in July. However, we only experimented for six days due to the limited number of the 5^th^ to 7^th^ leaves from the uppermost leaf, so we cannot be certain of survival rates of *S. exigua* throughout the entire development period.

**Figure 6:**
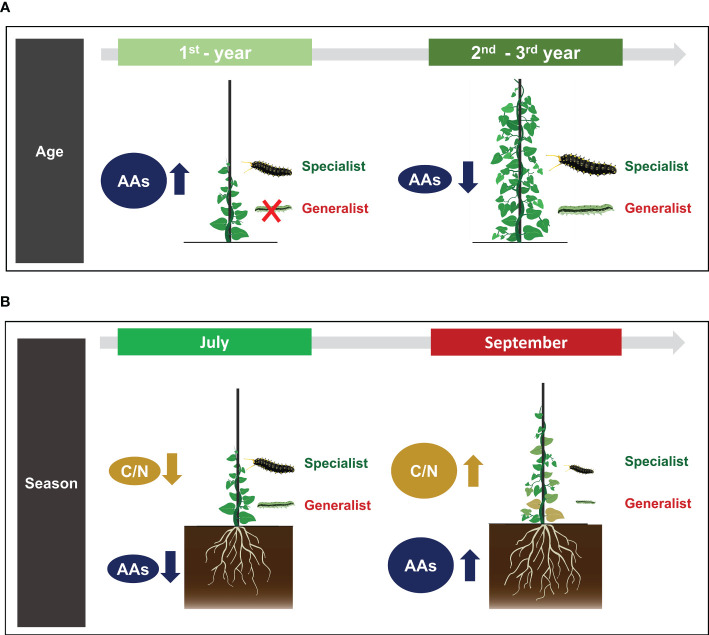
Summary of differences in secondary metabolite level of *Aristolochia contorta* according to plant age and season and their effects on performance of herbivorous insects: **(A)** 1^st^-year *A. contorta* invested more in chemical defense and effectively defended against generalist herbivore than specialist herbivore, **(B)** The higher C/N ratios of leaves in September than in July acted as a nutritional hurdle, suppressing the performance of surviving larvae. And root aristolochic acids concentrations became higher in the end of growing season than in the middle of growing season.

In conclusion, we evaluated how leaf-chewing specialist and generalist herbivores differed in performance according to age-dependent defensive traits of perennial herbs. Results showed that plant defense against herbivory stress might vary depending on plant age and season and that age-dependent defensive traits of plant could differently affect performances of specialist and generalist herbivores (**Figure 6**). *Aristolochia contorta* showed significantly higher concentrations of aristolochic acids in 1^st^-year leaves. Such high concentration seemed to effectively contribute to chemical defense against leaf-chewing generalist herbivore. Thus, it is presumed that *A. contorta* has a life history that mainly invests in chemical defense as its main defense mechanism to minimize damage from leaf-chewing herbivores in a young stage. In addition, the higher C/N ratios of leaves in September (the end of the growing season) than in July (the growing season) acted as a nutritional hurdle, suppressing the performance of surviving larvae. However, as emphasized earlier, *A. contorta* has the potential to invest more defense in its roots, including higher concentrations of aristolochic acids in roots than in leaves and increasing these concentrations with the passage of season. In particular, after 1^st^-year, *A. contort*a seems to give up the constitutive chemical aboveground defense and maintain only the belowground defense. Therefore, root defense of perennial herbs need to be further studied to obtain an in-depth understanding of ontogenetic trajectories of plant defense. The ontogenetic defense trajectories of perennial herbs we found in this study were different from studies that revealed that secondary metabolites generally increased across ontogeny in herbs (Barton and Koricheva, 2010; Quintero and Bowers, 2012; Kariñho-Betancourt et al., 2015). Indeed, the trajectory of ontogenetic chemical defense in herbs is species-specific and also can be dependent on the type of secondary metabolites (Popović et al., 2020; Yang et al., 2020). Therefore, the defense trajectories of these perennial herbs cannot be generalized to simplistic conclusions. Our findings contribute to the understanding of plant-herbivore interaction dynamics by suggesting that defense traits along the life history of perennial herbs can have differential effects on the performances of herbivores with different diet breadth.

## Data availability statement

The original contributions presented in the study are included in the article/**Supplementary Material**. Further inquiries can be directed to the corresponding author.

## Author contributions

JGK contributed to conceptualization and supervision of this study. SJJ, BEN, HJJ, JYJ and YJ performed experiments. SJJ and HJJ performed formal analysis. SJJ conducted visualization and wrote the first draft of the manuscript. BEN and JGK mainly edited the manuscript. All authors contributed to manuscript revision, read, and approved the submitted version. All authors contributed to the article and approved the submitted version.

## References

[B1] Aerts R. (1996). Nutrient resorption from senescing leaves of perennials: are there general patterns? J. Ecol. 84, 597–608. doi: 10.2307/2261481

[B2] Agrawal A. A. (2007). Macroevolution of plant defense strategies. Trends Ecol. Evol. 22, 103–109. doi: 10.1016/j.tree.2006.10.012 17097760

[B3] Alba C. Bowers M. D. Hufbauer R. (2012). Combining optimal defense theory and the evolutionary dilemma model to refine predictions regarding plant invasion. Ecology 93, 1912–1921. doi: 10.1890/11-1946.1 22928419

[B4] Ali J. G. Agrawal A. A. (2012). Specialist versus generalist insect herbivores and plant defense. Trends Plant Sci. 17, 293–302. doi: 10.1016/j.tplants.2012.02.006 22425020

[B5] Alves-Silva E. Del-Claro K. (2016). Herbivory-induced stress: leaf developmental instability is caused by herbivore damage in early stages of leaf development. Ecol. Indic. 61, 359–365. doi: 10.1016/j.ecolind.2015.09.036

[B6] Araya M. García S. González-Teuber M. (2018). Rapid identification and simultaneous quantification of aristolochic acids by HPLC-DAD and confirmations by MS in aristolochia chilensis using a limited biomass. J. Anal. Methods Chem. 2018, 1–8. doi: 10.1155/2018/5036542 PMC6011054 29977642

[B7] Barton K. E. Boege K. (2017). Future directions in the ontogeny of plant defence: understanding the evolutionary causes and consequences. Ecol. Lett. 20, 403–411. doi: 10.1111/ele.12744 28145095

[B8] Barton K. E. Koricheva J. (2010). The ontogeny of plant defense and herbivory: characterizing general patterns using meta-analysis. Am. Nat. 175, 481–493. doi: 10.1086/650722 20170370

[B9] Boege K. (2005). Herbivore attack in *Casearia nitida* influenced by plant ontogenetic variation in foliage quality and plant architecture. Oecologia 143, 117–125. doi: 10.1007/s00442-004-1779-9 15742219

[B10] Boege K. Barton K. E. Dirzo R. (2011). “Influence of tree ontogeny on plant-herbivore interactions,” in Size- and age-related changes in tree structure and function. Eds. Meinzer F. C. Lachenbruch B. Dawson T. E. (Dordrecht, CA: Springer), 193–214. doi: 10.1007/978-94-007-1242-3_7

[B11] Bryant J. P. Provenza F. D. Pastor J. Reichardt P. B. Clausen T. P. du Toit J. T. (1991). Interactions between woody plants and browsing mammals mediated by secondary metabolites. Annu. Rev. Ecol. Syst. 22, 431–446. doi: 10.1146/annurev.es.22.110191.002243

[B12] Cao H. H. Zhang Z. F. Wang X. F. Liu T. X. (2018). Nutrition versus defense: why *Myzus persicae* (green peach aphid) prefers and performs better on young leaves of cabbage. PloS One 13 (4), e0196219. doi: 10.1371/journal.pone.0196219 29684073 PMC5912751

[B13] Cheung T. P. Xue C. Leung K. Chan K. Li C. G. (2006). Aristolochic acids detected in some raw Chinese medicinal herbs and manufactured herbal products–a consequence of inappropriate nomenclature and imprecise labelling? Clin. Toxicol. 44, 371–378. doi: 10.1080/15563650600671712 16809138

[B14] Coley P. D. (1983). Herbivory and defensive characteristics oftree species in a lowland tropical forest. Ecol. Monogr. 53, 209–233. doi: 10.2307/1942495

[B15] Coley P. D. Bryant J. P. Chapin F. S. III (1985). Resource availability and plant antiherbivore defense. Science 230, 895–899. doi: 10.1126/science.230.4728.895 17739203

[B16] Cope O. L. Burkle L. A. Croy J. R. Mooney K. A. Yang L. H. Wetzel W. C. (2022). The role of timing in intraspecific trait ecology. Trends Ecol. Evol. 37, 997–1005. doi: 10.1016/j.tree.2022.07.003 35918208

[B17] Couture J. J. Servi J. S. Lindroth R. L. (2010). Increased nitrogen availability influences predator–prey interactions by altering host-plant quality. Chemoecology 20, 277–284. doi: 10.1007/s00049-010-0058-y

[B18] Cui X. Meng F. Pan X. Qiu X. Zhang S. Li C.. (2022). Chromosome-level genome assembly of *Aristolochia contorta* provides insights into the biosynthesis of benzylisoquinoline alkaloids and aristolochic acids. Hortic. Res. 9, uhac005. doi: 10.1093/hr/uhac005 35147168 PMC8973263

[B19] Dirzo R. Mendoza E. Ortíz P. (2007). Size-related differential seed predation in a heavily defaunated neotropical rain forest. Biotropica 39, 355–362. doi: 10.1111/j.1744-7429.2007.00274.x

[B20] Ernest K. A. (1989). Insect herbivory on a tropical understory tree: effects of leaf age and habitat. Biotropica 21, 194–199. doi: 10.2307/2388642

[B21] Fischer K. Fiedler K. (2000). Response of the copper butterfly *Lycaena tityrus* to increased leaf nitrogen in natural food plants: evidence against the nitrogen limitation hypothesis. Oecologia 124, 235–241. doi: 10.1007/s004420000365 28308184

[B22] Fordyce J. A. Marion Z. H. Shapiro A. M. (2005). Phenological variation in chemical defense of the pipevine swallowtail, *Battus philenor* . J. Chem. Ecol. 31, 2835–2846. doi: 10.1007/s10886-005-8397-9 16365708

[B23] Franklin O. Agren G. I. (2002). Leaf senescence and resorption as mechanisms of maximizing photosynthetic production during canopy development at n limitation. Funct. Ecol. 16, 727–733. doi: 10.1046/j.1365-2435.2002.00674.x

[B24] Goodger J. Q. Heskes A. M. Woodrow I. E. (2013). Contrasting ontogenetic trajectories for phenolic and terpenoid defences in *Eucalyptus froggattii* . Ann. Bot. 112, 651–659. doi: 10.1093/aob/mct010 23378522 PMC3736765

[B25] Gutbrodt B. Dorn S. Unsicker S. B. Mody K. (2012). Species-specific responses of herbivores to within-plant and environmentally mediated between-plant variability in plant chemistry. Chemoecology 22, 101–111. doi: 10.1007/s00049-012-0102-1

[B26] Habekost M. Eisenhauer N. Scheu S. Steinbeiss S. Weigelt A. Gleixner G. (2008). Seasonal changes in the soil microbial community in a grassland plant diversity gradient four years after establishment. Soil Biol. Biochem. 40, 2588–2595. doi: 10.1016/j.soilbio.2008.06.019

[B27] Han P. Lavoir A. V. Le Bot J. Amiens-Desneux E. Desneux N. (2014). Nitrogen and water availability to tomato plants triggers bottom-up effects on the leafminer *Tuta absoluta* . Sci. Rep. 4, 1–8. doi: 10.1038/srep04455 PMC5380111 24675796

[B28] Hanley M. E. Fegan E. L. (2007). Timing of cotyledon damage affects growth and flowering in mature plants. Plant Cell Environ. 30, 812–819. doi: 10.1111/j.1365-3040.2007.01671.x 17547653

[B29] Herms D. A. Mattson W. J. (1992). The dilemma of plants: to grow or defend. Q. Rev. Biol. 67, 283–335. doi: 10.1086/417659

[B30] Huot B. Yao J. Montgomery B. L. He S. Y. (2014). Growth–defense tradeoffs in plants: a balancing act to optimize fitness. Mol. Plant 7, 1267–1287. doi: 10.1093/mp/ssu049 24777989 PMC4168297

[B31] Iqbal Z. Iqbal M. S. Hashem A. Abd_Allah E. F. Ansari M. I. (2021). Plant defense responses to biotic stress and its interplay with fluctuating dark/light conditions. Front. Plant Sci. 12. doi: 10.3389/fpls.2021.631810 PMC7982811 33763093

[B32] Iwasa Y. (2000). Dynamic optimization of plant growth. Evol. Ecol. Res. 2 (4), 437–455.

[B33] Jeude S. E. Fordyce J. A. (2014). The effects of qualitative and quantitative variation of aristolochic acids on preference and performance of a generalist herbivore. Entomol. Exp. Appl. 150, 232–239. doi: 10.1111/eea.12159

[B35] Karban R. Agrawal A. A. (2002). Herbivore offense. Annu. Rev. Ecol. Syst. 33, 641–664. doi: 10.1146/annurev.ecolsys.33.010802.150443

[B34] Karban R. Myers J. H. (1989). Induced plant responses to herbivory. Annu. Rev. Ecol. Syst. 20, 331–348. doi: 10.1146/annurev.es.20.110189.001555

[B36] Kariñho-Betancourt E. Agrawal A. A. Halitschke R. Núñez-Farfán J. (2015). Phylogenetic correlations among chemical and physical plant defenses change with ontogeny. New Phytol. 206, 796–806. doi: 10.1111/nph.13300 25652325

[B37] Kearsley M. J. C. Whitham T. G. (1998). The developmental stream of cottonwoods affects ramet growth and resistance to galling aphids. Ecology 79, 178–191. doi: 10.1890/0012-9658(1998)079[0178:tdsoca]2.0.co;2

[B38] Kim D. S. Kwon Y. J. (2010). Metapopulation dynamics of the oriental long-tailed swallow *Sericinus montela* (Lepidiptera: papilionidae) in Korea. Kor J. Appl. Entomol. 49, 289–297. doi: 10.5656/ksae.2010.49.4.289

[B39] Kuglerová M. Skuhrovec J. Münzbergová Z. (2019). Relative importance of drought, soil quality, and plant species in determining the strength of plant–herbivore interactions. Ecol. Entomol. 44, 665–677. doi: 10.1111/een.12745

[B40] Lankau R. A. (2007). Specialist and generalist herbivores exert opposing selection on a chemical defense. New Phytol. 175, 176–184. doi: 10.1111/j.1469-8137.2007.02090.x 17547677

[B41] Li H. Crabbe M. J. C. Xu F. Wang W. Niu R. Gao X.. (2017). Seasonal variations in carbon, nitrogen and phosphorus concentrations and C:N:P stoichiometry in the leaves of differently aged larix principis-rupprechtii mayr. plantations. Forests 8, 373. doi: 10.3390/f8100373 PMC5609765 28938020

[B42] Maron J. L. Agrawal A. A. Schemske D. W. (2019). Plant–herbivore coevolution and plant speciation. Ecology 100, e02704. doi: 10.1002/ecy.2704 30916391

[B43] Mattson W. J. (1980). Herbivory in relation to plant nitrogen content. Annu. Rev. Ecol. Syst. 11, 119–161. doi: 10.1146/annurev.es.11.110180.001003

[B44] McKey D. (1974). Adaptive patterns in alkaloid physiology. Am. Nat. 108, 305–320. doi: 10.1086/282909

[B46] Moura J. C. M. S. Bonine C. A. V. de Oliveira Fernandes Viana J. Dornelas M. C. Mazzafera P. (2010). Abiotic and biotic stresses and changes in the lignin content and composition in plants. J. Integr. Plant Biol. 52, 360–376. doi: 10.1111/j.1744-7909.2010.00892.x 20377698

[B47] Nakonechnaya O. V. Kholina A. B. Koren O. G. Zhuravlev Y. N. (2012). Genetic diversity of a rare species *Aristolochia contorta* bunge (Aristolochiaceae) in primorsky krai. Russ. J. Genet. 48, 152–162. doi: 10.1134/s1022795411120088 22567996

[B48] Nam B. E. Park H. J. Son G. Y. Kim J. G. (2020). An analysis of the genetic diversity of a riparian marginal species, *Aristolochia contorta* . J. Wet. Res. 22, 100–105. doi: 10.17663/JWR.2020.22.2.100

[B49] National Institute of Biological Resources (2012). Red data book of endangered insects in Korea I (Seoul: Ministry of Environment).

[B51] Ochoa-López S. Damián X. Rebollo R. Fornoni J. Domínguez C. A. Boege K. (2020). Ontogenetic changes in the targets of natural selection in three plant defenses. New Phytol. 226, 1480–1491. doi: 10.1111/nph.16422 31943211

[B52] Ohnmeiss T. E. Baldwin I. T. (2000). Optimal defense theory predicts the ontogeny of an induced nicotine defense. Ecology 81, 1765–1783. doi: 10.1890/0012-9658(2000)081[1765:odtpto]2.0.co;2

[B55] Park S. H. Nam B. E. Kim J. G. (2019). Shade and physical support are necessary for conserving the *Aristolochia contorta* population. Ecol. Eng. 135, 108–115. doi: 10.1016/j.ecoleng.2019.05.019

[B53] Park H. J. Nam B. E. Lee G. Kim S. G. Joo Y. Kim J. G. (2022). Ontogeny-dependent effects of elevated CO_2_ and watering frequency on interaction between *Aristolochia contorta* and its herbivores. Sci. Total Environ. 838, 156065. doi: 10.1016/j.scitotenv.2022.156065 35597357

[B54] Park H. J. Nam B. E. Moon S. Y. Kim S. G. Joo Y. Kim J. G. (2021). Reduced host plant growth and increased tyrosine-derived secondary metabolites under climate change and negative consequences on its specialist herbivore. Sci. Total Environ. 759, 143507. doi: 10.1016/j.scitotenv.2020.143507 33223185

[B500] Pérez-Harguindeguy N. Diaz S. Vendramini F. Cornelissen J. H. C. Gurvich D. E. Cabido M. (2003). Leaf traits and herbivore selection in the field and in cafeteria experiments. Austral Ecol. 28, 642–650. doi: 10.1046/j.1442-9993.2003.01321.x

[B56] Popović Z. Milošević D. K. Stefanović M. Vidaković V. Matić R. Janković J.. (2020). Variability of six secondary metabolites in plant parts and developmental stages in natural populations of rare *Gentiana pneumonanthe* . Plant Biosyst. 155, 816–822. doi: 10.1080/11263504.2020.1785966

[B57] Priestap H. A. Velandia A. E. Johnson J. V. Barbieri M. A. (2012). Secondary metabolite uptake by the *Aristolochia*-feeding papilionoid butterfly *Battus polydamas* . Biochem. Syst. Ecol. 40, 126–137. doi: 10.1016/j.bse.2011.10.006

[B58] Quintero C. Bowers M. D. (2012). Changes in plant chemical defenses and nutritional quality as a function of ontogeny in *Plantago lanceolata* (Plantaginaceae). Oecologia 168, 471–481. doi: 10.1007/s00442-011-2114-x 21913028

[B59] Royer M. Larbat R. Le Bot J. Adamowicz S. Robin C. (2013). Is the C:N ratio a reliable indicator of c allocation to primary and defence-related metabolisms in tomato? Phytochemistry 88, 25–33. doi: 10.1016/j.phytochem.2012.12.003 23312460

[B60] Saska P. Skuhrovec J. Tylová E. Platková H. Tuan S.-J. Hsu Y.-T.. (2021). Leaf structural traits rather than drought resistance determine aphid performance on spring wheat. J. Pest. Sci. 94, 423–434. doi: 10.1007/s10340-020-01253-3

[B61] Schneider C. A. Rasband W. S. Eliceiri K. W. (2012). NIH Image to ImageJ: 25 years of image analysis. Nat. Methods 9, 671–675. doi: 10.1038/nmeth.2089 22930834 PMC5554542

[B62] Shin Y. H. (1974). Life history of sericinus telamon Donovan in Korea (Seoul: Kyung Hee University).

[B63] Sime K. R. Feeny P. P. Haribal M. M. (2000). Sequestration of aristolochic acids by the pipevine swallowtail, *Battus philenor* (L.): evidence and ecological implications. Chemoecology 10, 169–178. doi: 10.1007/pl00001819

[B64] Simms E. L. Rausher M. D. (1987). Costs and benefits of plant resistance to herbivory. Am. Nat. 130, 570–581. doi: 10.1086/284731

[B65] Strauss S. Y. Agrawal A. A. (1999). The ecology and evolution of plant tolerance to herbivory. Trends Ecol. Evol. 14, 179–185. doi: 10.1016/s0169-5347(98)01576-6 10322530

[B66] Thiel T. Gaschler S. Mody K. Blüthgen N. Drossel B. (2020). Impact of plant defense level variability on specialist and generalist herbivores. Theor. Ecol. 13, 409–424. doi: 10.1007/s12080-020-00461-y

[B67] Traw M. B. Feeny P. (2008). Glucosinolates and trichomes track tissue value in two sympatric mustards. Ecology 89, 763–772. doi: 10.1890/07-0729.1 18459339

[B68] Yang L. H. Cenzer M. L. Morgan L. J. Hall G. W. (2020). Species-specific, age-varying plant traits affect herbivore growth and survival. Ecology 101, e03029. doi: 10.1101/2020.01.14.906859 32115691

