# Supplementary material for: Age-dependent resistance of a perennial herb, Aristolochia contorta against specialist and generalist leaf-chewing herbivores
Source: Front Plant Sci. 2023 May 30;14:1145363. doi: 10.3389/fpls.2023.1145363 (PMC10265686; doi:10.3389/fpls.2023.1145363)
Supplement: Supplementary file 1 [file DataSheet_1.docx]

Supplementary Material

Age-dependent resistance of a perennial herb, *Aristolochia contorta* against specialist and generalist leaf-chewing herbivores

Se Jong Jeong, Bo Eun Nam, Hyeon Jin Jeong, Jae Yeon Jang, Youngsung Joo, Jae Geun Kim*

*** Correspondence:** jaegkim@snu.ac.kr

## Supplementary Figures


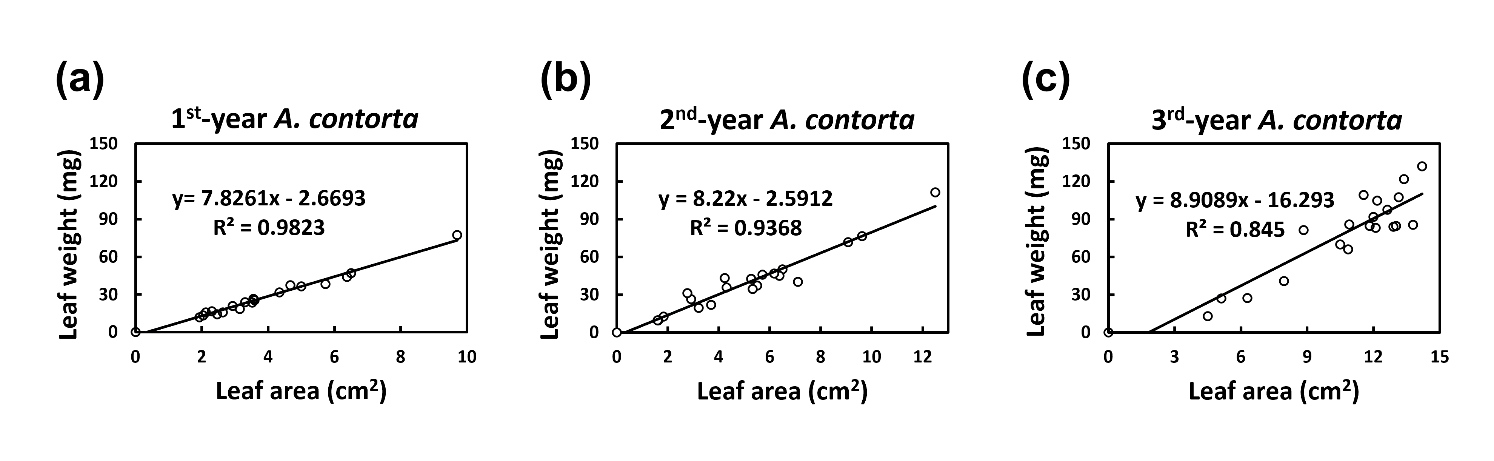


**Figure S1.** Formulae on the leaf weight from leaf area of *A*. *contorta* for each age group. **(a)** 1^st^-year *A. contorta* leaves, **(b)** 2^nd^-year *A. contorta* leaves, **(c)** 3^rd^-year *A. contorta* leaves, where *x* was the leaf area (cm^2^), *y* was the leaf weight (mg). Constants were estimated from subsampled *A. contorta* leaves according to plant age (*n* = 20, respectively).


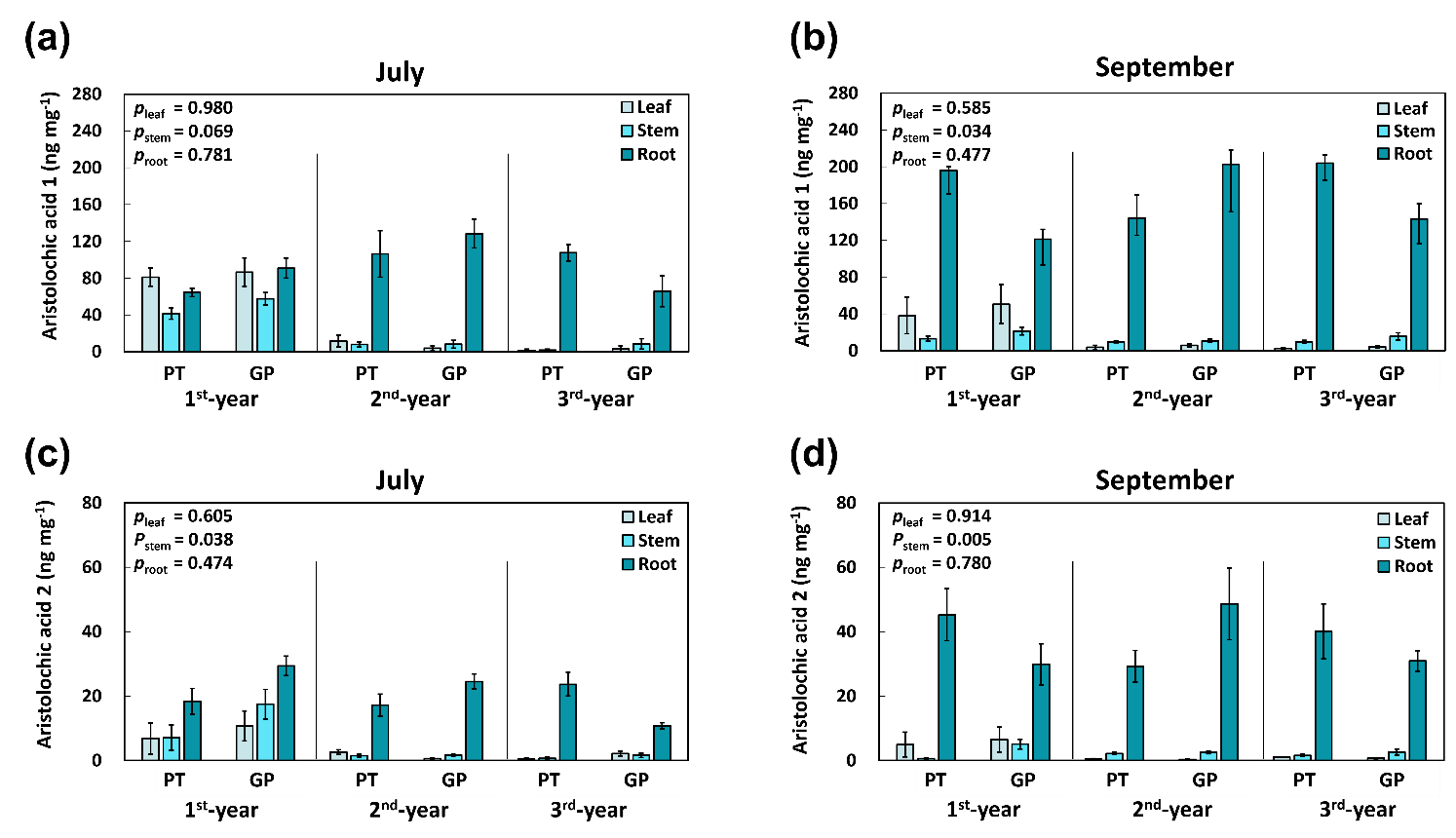


**Figure S2.** Aristolochic acids concentrations in each of the plant leaves and roots were different with plant age, not by source populations. *p* values refer the significance of the origin population (*df* = 1) from one-way ANOVA. **(a, b)** Aristolochic acid 1, **(c, d)** Aristolochic acid 2. PT, Pyeongtaek (*n* = 5 for July; *n* = 5 for September, in case of 1^st^-year roots (September), *n* = 4); GP, Gapyeong (*n* = 5 for July; *n* = 5 for September).

##
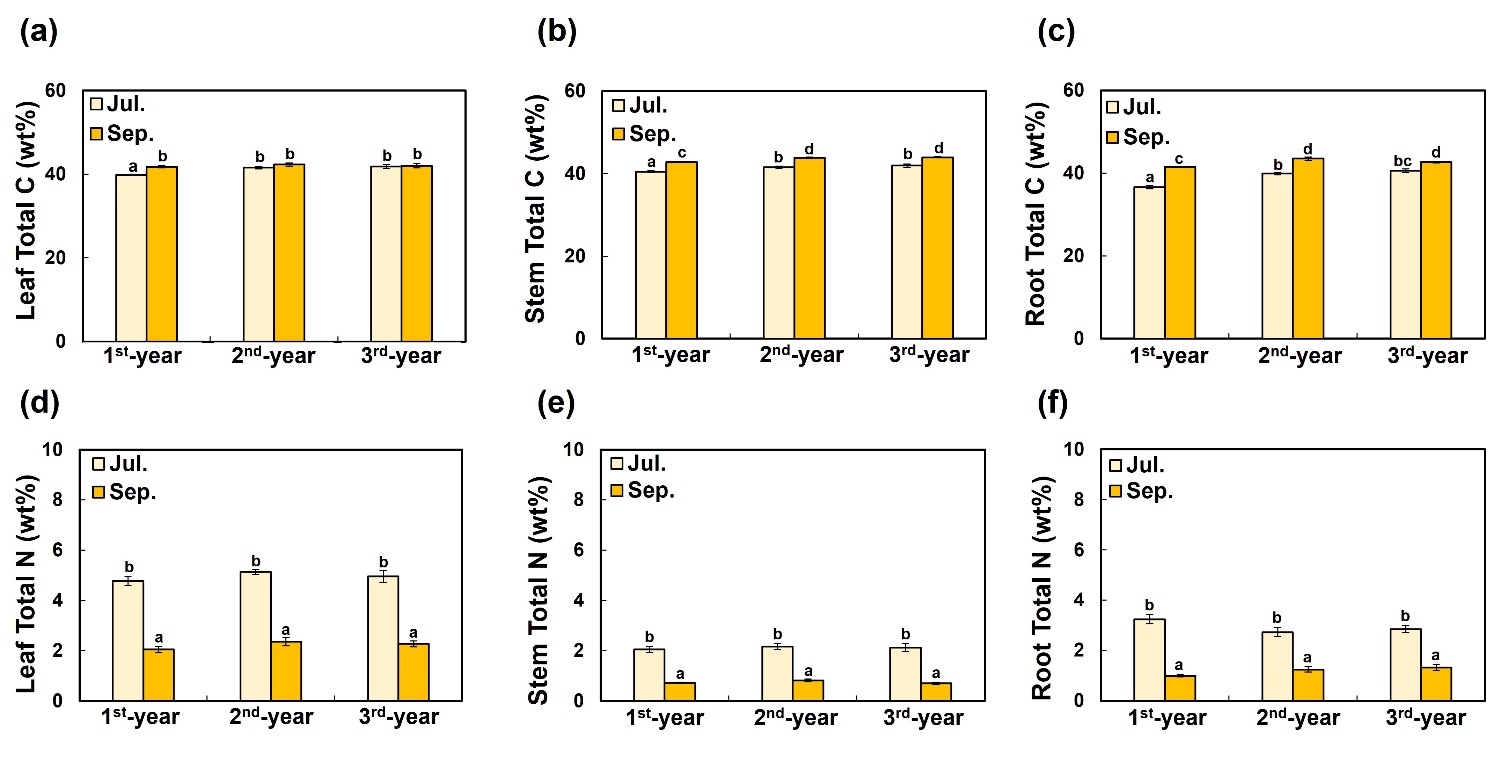


**Figure S3.** The total C and N (wt%) of each organ of the plants. **(a)** leaf total C , **(b)** stem total C, **(c)** root total C, **(d)** leaf total N, **(e)** stem total N, **(f)** root total N. The vertical bars show the standard error for each group (*n* = 7, respectively). Different letters represent statistically different sub-groups by Duncan's post-hoc test (*p* < 0.05).


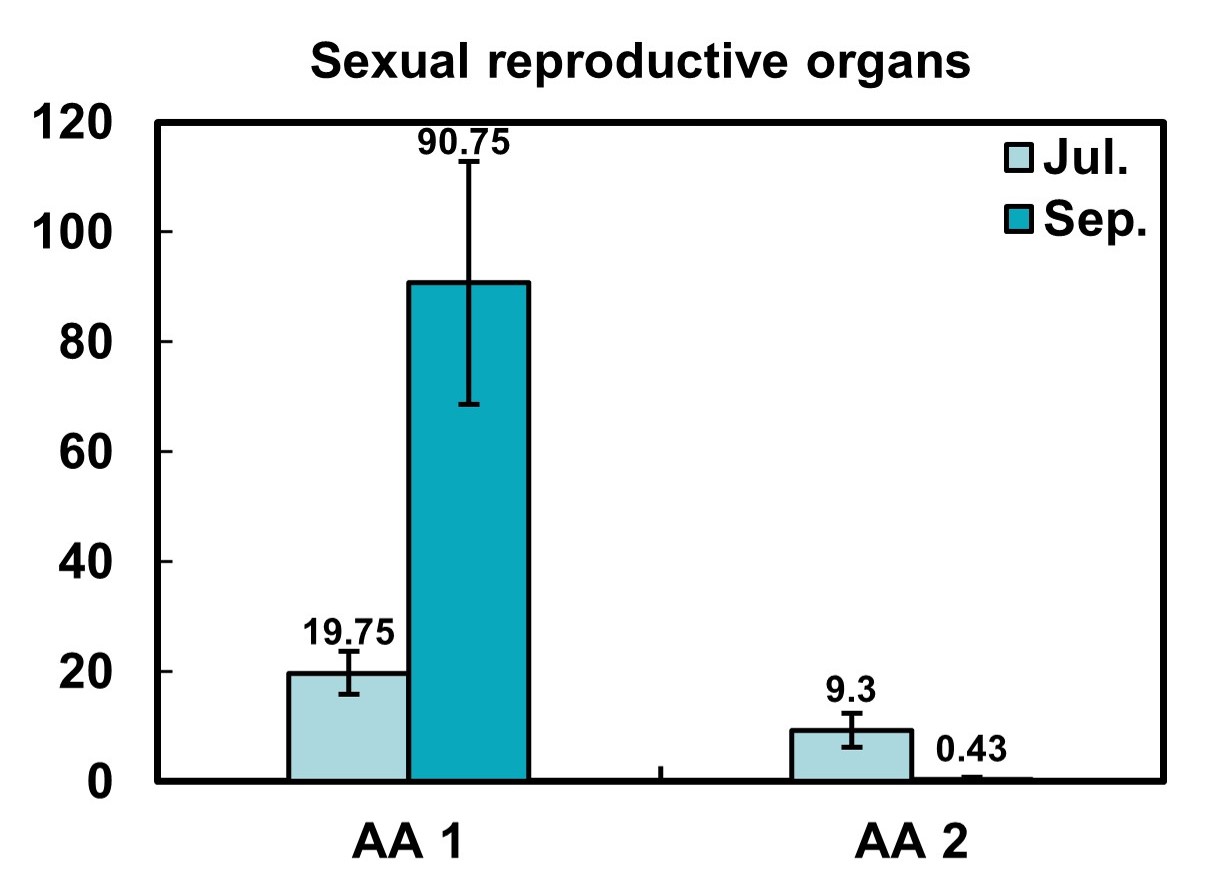


## Figure S4. The aristolochic acid 1(AA 1) and aristolochic acid 2(AA 2) contents in Sexual reproductive organs of 3rd-year *A.contorta*. This content of the sexual reproductive organs was lower than that of the root. The vertical bars show the standard error for each group (*n* = 8 in Jun, *n* = 5 in Sep).
